# Toward Customizable Smart Gels: A Comprehensive Review of Innovative Printing Techniques and Applications

**DOI:** 10.3390/gels11010032

**Published:** 2025-01-02

**Authors:** Rizwan Ul Hassan, Naseem Abbas, Jongkuk Ko

**Affiliations:** 1 School of Chemical, Biological, and Battery Engineering, Gachon University, 1342 Seongnam-daero, Seongnam-si 13120, Republic of Korea; rizwanhassan72@gachon.ac.kr; 2 Department of Mechanical Engineering, Sejong University, Gwangjin-gu, Seoul 05006, Republic of Korea

**Keywords:** intelligent structures, fabrication techniques, smart materials

## Abstract

New production technologies have transformed modern engineering fields, including electronics, mechanics, robotics, and biomedicine. These advancements have led to the creation of smart materials such as alloys, polymers, and gels that respond to various stimuli. This review focuses on smart materials (SMs), including their variety and fabrication techniques, that can be used to construct three- or four-dimensional structures. The mechanisms and designs of smart materials, limitations of current printing technologies, and perspectives for their future uses are also discussed in this review. The printed smart materials are expected to have a major impact on the design of real-world applications.

## 1. Introduction

Modern living conditions have greatly improved because of the transformation of several fields and the development of relatively inexpensive materials through technological and scientific advancements and the ongoing expansion of contemporary technology. Such advancements include the development of new materials and synthesis methods, as well as characterization and printing techniques, creating a new paradigm for numerous innovative applications [1]. Materials (alloys, ceramics, metals, polymers, gels, composites, and smart materials) used in different additive manufacturing (AM) technologies can be categorized according to their mechanical, chemical, and physical characteristics. Smart materials (smart alloys, polymers, and gels) exhibit characteristics that can be altered in response to external stimuli such as pH, light, heat, water, or electromagnetic fields.

Smart materials have been a hot research topic for many years. Olander first identified smart alloys (SAs) or shape memory alloys (SMAs) in 1932 [2], whereas the term “shape memory” was first coined by Vernon in 1954. The importance of shape memory materials (SMMs) was recognized when researchers discovered the shape memory effect (SME) in a nickel–titanium (NiTi) alloy in 1962, in which the transition was driven by temperature [3]. Since then, various commercial fields have employed SMMs in the construction of structures and composites for industrial applications [4,5]. These fields include the automotive [6], aerospace [7], biomedical [8], robotics [9], and fashion [10] industries. The demand for technical and engineering applications has led to an increasing need for copper and iron shape memory alloys (SMAs), which offer high and comparable resolutions and are inexpensive and widely available commercially [11]. However, due to their greater stability, resilience, and superior thermomechanical performance [12], NiTi-based SMAs are preferred, despite their high cost. Nevertheless, each material offers distinct advantages for specific requirements and applications. SMAs are categorized according to their application domain, including automotive, aerospace, robotics, and biomedical applications.

Other than SMAs, shape memory polymers (SMPs), also referred to as smart polymers, represent a category of intelligent materials that offer rapid programming capabilities and can be adapted for a diverse array of applications. SMPs are often considered preferable alternatives to SMAs because of their low cost, enhanced efficiency, biodegradability, and superior mechanical properties [13,14]. Moreover, SMPs can undergo multiple shape changes [15] triggered by various external stimuli, such as thermal conditions (heating [16] or cooling [17]), electricity [18], magnetic fields [19], light [20], or specific solutions (chemicals or water [21]). Generally, SMPs can be classified into three main types [22], which are inherently responsive to thermal and chemical stimuli. Polymer-based products have a wide array of commercial applications, highlighting the significant potential of SMPs [23]. These applications include smart fabrics and self-repairing or -seal-healing plastic components [24], spacecraft components [25], biomedical devices [26], and intelligent structures. The SME in polymeric materials functions through three fundamental mechanisms: the dual-state mechanism, dual-component mechanism (DCM), and partial transition mechanism (PTM) [21]. SMPs are well suited for demanding applications, affording a recovery precision of over 99%. The SMEs in SMPs and SMAs vary depending on factors such as the material composition, weight fraction of switching segments, and molecular weight of the polymer chain. Some SMPs offer the advantage of biodegradability, allowing implants to be removed after regeneration, and enhancing the feasibility, effectiveness, and cost efficiency of therapies compared to metal implants. Despite these advantages, SMAs remain the preferred material for applications that require higher actuation pressures and faster response times.

The demand for smart materials with novel physical properties (such as elasticity, high water absorption, softness, and low friction) to meet the emerging requirements of both engineering and medical applications is constantly increasing, given the soft and wet nature of these materials. Therefore, numerous researchers have also developed gel materials in addition to SMAs and SMPs [27]. Shape memory gels (SMGs) are one of the most intriguing new smart materials because of their biocompatibility and biodegradability. When heated above the critical temperature, SMGs undergo a phase change, soften, and become elastic. SMGs can return to their former shape during the gelation process; this is known as shape recovery. SMGs are appropriate for various applications, including bandages for fractured bones [28], the creation of optical lenses for eyeballs [29], and the development of smart devices [30].

These smart materials are printed by different printing techniques, and are particularly important in three-dimensional (3D) and four-dimensional (4D) printings, where they are used to print objects that can undergo temporal-dependent shape changes. Three-dimensional printing is considered an innovative and growing production process for many materials and is already being implemented at an industrial level [31]. Three-dimensional printing is also gaining popularity owing to its capacity for printing complicated shapes and parts with high accuracy, global availability, and versatility [32]. Several 3D printing technologies, such as laser, ink, and light-based [33] methods, are effective for a wide range of materials, including polymers, elastomers, metals, and polymer composites. Three-dimensional printing offers several advantages in terms of sustainability, because diverse natural biomaterials can be used as ink sources for new applications, ensuring minimal or no waste generation. Additionally, 3D printing enhances the mechanical properties of composite materials beyond those of traditional composites [34]. The capability for multimaterial printing has further enabled the creation of complex structures such as helical coils and functionalized microarchitectures, with enhanced precision. Three-dimensional printing has also opened up numerous fascinating avenues for actual applications and continues to flourish as a method for incorporating various developing materials, including smart materials, to achieve broad community goals [35].

During the coronavirus (COVID-19) pandemic, personal protective equipment was fabricated by 3D printing [36]. Its other biomedical applications include printing patient-specific models for training medical staff, wearable devices such as orthotics and prosthetics [37], tissue engineering [38], drug delivery systems [39], and life-enhancing gadgets [40]. Three-dimensional printing is becoming more prevalent in various disciplines and systems, including analytical chemistry, microfluidic devices, and medical diagnostics for analyte detection, electrochemical sensors, and health monitoring systems. However, challenges such as high costs, limited availability of printing materials, the necessity of post-processing of printed devices, and the quest for improved resolution continue to hinder the widespread adoption of this technique. A significant drawback is the static nature of the printed functionalities post-3D printing, which limits their application in emerging fields where dynamic capabilities such as self-healing, conductivity in the stretched state, and shape-morphing functions are essential (e.g., wearable electronics, soft robotics, and flexible biosensors) [41]. Printed materials’ ability to change their shape is critical for advanced engineering applications [42]. Therefore, the 4D printing process, inspired by natural shape-morphing behaviors, has recently been developed as an enhanced form of AM [43]. Three-dimensional printing using smart materials that respond to external stimuli in a temporal manner forms the basis of 4D printing [44]. Four-dimensional printing technology enables more precise control over the shapes of the printed products, which can undergo shrinking, swelling, folding, bending, rolling, origami formation, twisting, and motility in response to various environmental stimuli.

This review provides an overview of smart materials (SMs), including their variety and fabrication techniques. The main focus is on printing methods for producing smart materials such as shape memory metals, polymers, and hydrogels. This review also demonstrates the potential of smart materials (particularly shape memory gels) through innovative printing techniques, focusing on their role in revolutionizing engineering and biomedical fields. In-depth analyses of the 3D and 4D printing technologies used to fabricate a variety of smart materials with unique responsive properties are described. Our comprehensive study highlights advancements in printing methods to create customizable, biocompatible, and biodegradable SMGs that play pivotal roles in various applications ranging from tissue engineering to soft robotics. The applications of printed SMs are divided into several categories based on the end use and other domains. Several current issues and emerging areas related to smart material printing technologies are also discussed, offering insights into the evolving landscape of smart material designs and applications.

## 2. Smart Materials

SMs are a class of materials that can return to their original shape after memorization through two transformation stages. This phenomenon is known as the SME, whereby a material undergoes deformation under an external force and subsequently returns to its original shape when heated above a specific temperature. This temperature elevation in SMEs can occur through external or internal heating, or can be triggered by other stimuli, such as pressure, electric fields, magnetic fields, pH changes, or exposure to water. Here, we present an in-depth discussion of the SMEs of different smart materials.

### 2.1. Shape Memory Effects in SMs

**Figure 1:**
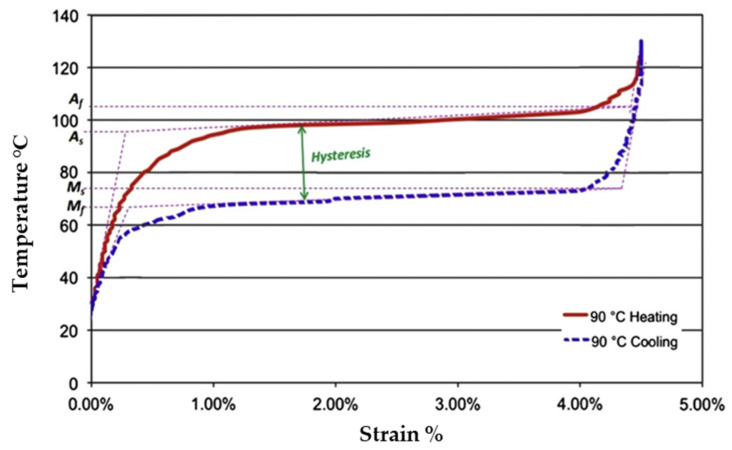
Hysteresis loop and phase transformation for shape memory materials. Adapted with permission from [47].

**Figure 2:**
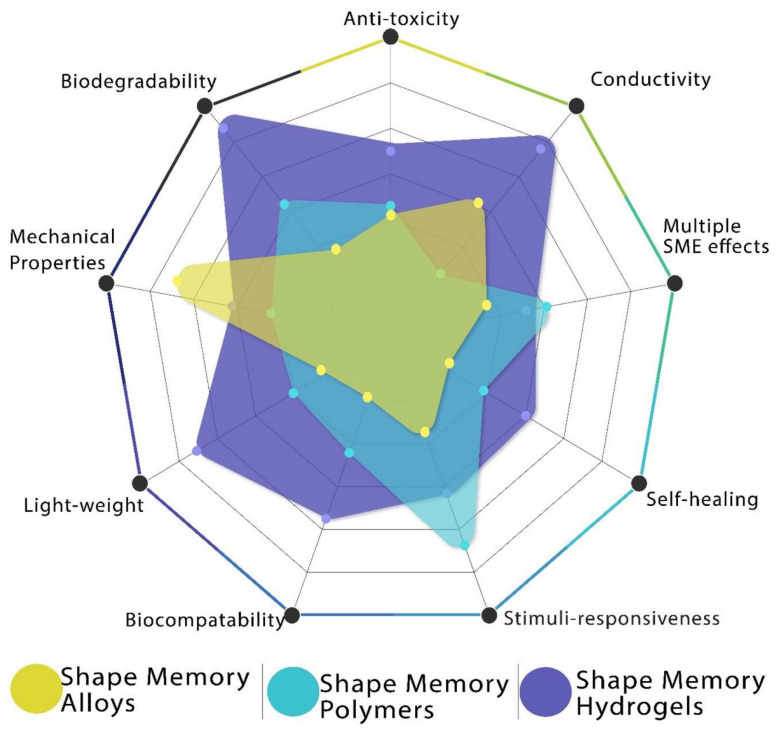
Comparison between the properties of shape memory materials. Adapted with permission from [50].

SMAs exhibit dual phases and can adopt three distinct crystal structures: twinned martensite, detwinned martensite, and austenite. These phases can undergo six possible transformations [45,46]. Austenite is stable at higher temperatures, whereas martensite is stable at lower temperatures. Upon heating, the SMAs undergo a transition from the martensite to the austenite phase. The austenite start temperature (A_s_) marks the onset of this transformation, whereas the austenite finish temperature (A_f_) indicates its completion. Heating beyond A_s_ triggers compression and transformation into an austenitic structure, enabling the material to revert to its original shape even under significant applied loads. These characteristics yield substantial actuation energy densities. During the cooling process, the transformation in SMAs begins at the martensite start temperature (M_s_) and ends at the martensite-finish temperature (M_f_) [47] (Figure 1). In Figure 1, the hysteresis loop shows the relationship between stress and strain during the material’s heating and cooling cycles, which is essential to comprehending the behavior of shape memory materials. The loop highlights the energy loss that occurs due to internal friction, where area within the loop represents the amount of energy dissipated during one cycle, which is important for evaluating the efficiency and performance of shape memory materials. The maximum temperature at which martensite can no longer be stressed is denoted as M_d_, above which the SMA undergoes irreversible deformation, similar to any other metallic material. Hysteresis, defined as the difference in the transition temperature during heating and cooling (DT = A_f_ − M_s_), typically represents the temperature at which the material transforms to 50% austenite upon heating and to 50% martensite upon cooling [48]. Hysteresis is a crucial factor in the selection of SMA materials for specific technical applications. For instance, rapid actuation necessitates minimal hysteresis. Similarly to SMAs, SMEs in SMPs vary based on the material composition, operating through three fundamental mechanisms [49], with a recovery precision exceeding 99%. Certain biodegradable SMPs offer advantages over metal implants by eliminating the need for implant removal after regeneration. Despite these advantages, SMAs remain superior for applications that require higher actuation pressures and faster response times. SMGs maintain their original shape during gelation, a phenomenon referred to as shape recovery. Upon heating beyond a critical temperature, the SMGs soften and become elastic, undergoing a phase change. SMGs can be restored to their initial shapes after deformation by reheating above this critical temperature. Figure 2 provides a detailed comparison of different shape memory materials, with further discussions on each material (SMAs, SMPs, SMGs) presented in the following sections [50].

### 2.2. Shape Memory Alloys

SMAs were first identified in 1932 [2] and solid-phase transformations were discovered in SMAs, where it was observed that gold–cadmium alloys could be plastically deformed during cooling and returned to their original shape when heated. Subsequently, in 1938, other researchers [51] identified the SMEs in copper–zinc and copper–tin alloys. The fundamental properties of SMEs are governed by the thermoelastic behavior of the martensite phase [52]. Similar behaviors have been observed in other alloys, including In-Tl and Cu-Al-Ni alloys. These discoveries sparked significant academic and industrial interest; however, the practical applications of SMAs have been hindered by high material costs, production complexity, and undesirable mechanical properties. It was not until the SME was demonstrated in the NiTi alloy [53], that the potential for commercial SMA applications was realized. Nitinol alloys have proven to be more economical to manufacture, easier to handle, and exhibit superior mechanical properties compared with other available SMAs. The first commercial success for SMA applications came with a “shrink-to-fit” pipe coupler for the F-14 jet fighter. Since then, the use of NiTi alloys in commercial applications has expanded across various industries due to the growing demand for lighter and more compact actuators, particularly in healthcare.

Recent developments in shape memory technology (SMT) have led to significant changes in SMAs applications and design, along with the widespread commercial use of SMAs in the automotive, aerospace, robotics, and biomedical fields. SMAs actuators have proven effective in applications involving low-frequency vibrations and actuation. Ongoing research efforts aim to systematically enhance the SMAs performance by focusing on improving features such as the bandwidth, fatigue life, and stability [54,55]. Recent experimental approaches include refining the compositions of materials to adjust the phase transition temperatures of SMAs [56,57], expanding the operational temperature ranges, and enhancing the material response and actuation strain through improved mechanical designs, control systems, and manufacturing processes. Besides SMAs, SMPs are also extensively studied by researchers, and will be further discussed in the context of shape memory materials.

### 2.3. Shape Memory Polymers

SMPs are advanced smart materials that can be bent and set into a temporary shape and can subsequently revert to their original shape upon removal of an external stimulus [58,59]. Their appealing attributes, including structural versatility, light weight, affordability, ease of processing, high elastic strain capacity, biocompatibility, and biodegradability, have propelled their adoption in diverse industrial sectors such as aerospace, textiles, and medicine [60,61]. SMPs can respond to external triggers such as heat, light, electricity, magnetic fields, chemical stimuli, and humidity.

Recent reviews on SMPs have explored their activation mechanisms, molecular features, and changes during activation of the SME, as well as their wide-ranging applications, including aerospace engineering, sensors and actuators, textile engineering, artificial muscles, and packaging [62,63,64,65]. SMPs are particularly suitable for smart systems that require multiple functionalities and are responsive to diverse external inputs, because they can retain more than two temporary shapes during their SME cycles. However, compared to well-established shape memory alloys, SMPs have limitations such as low thermal and electrical conductivity, poor mechanical strength, and low responsiveness to electromagnetic stimuli [66].

**Figure 3:**
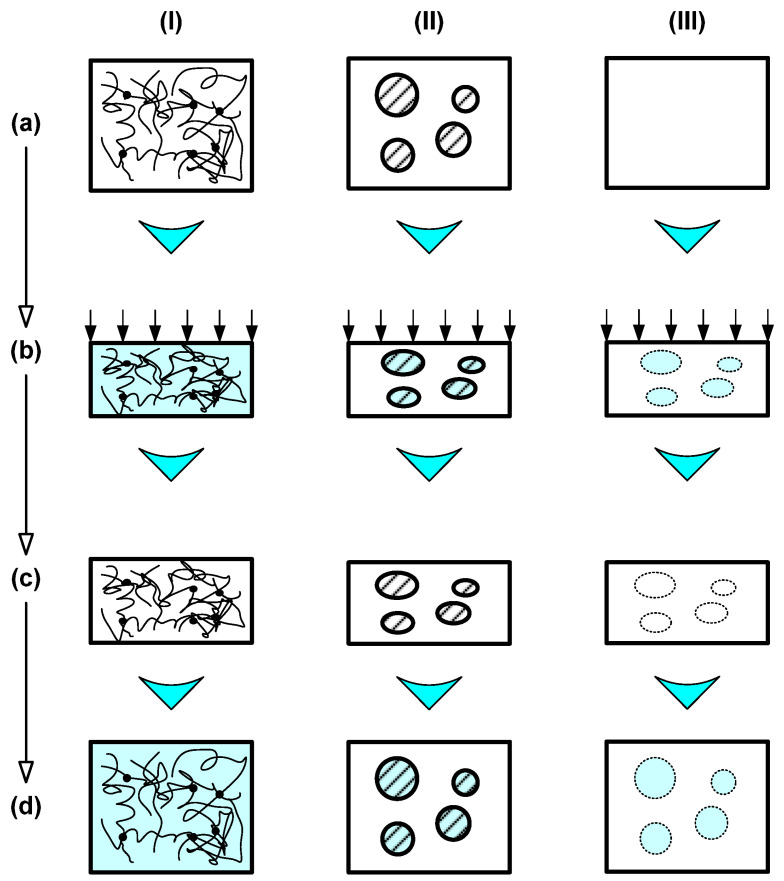
Basic working mechanisms for the heating-responsive SME in polymeric materials. (**I**) Dual-state mechanism (DSM); (**II**) dual-component mechanism (DCM); (**III**) partial-transition mechanism (PTM). (**a**) Original sample at low temperatures; (**b**) upon heating and compressing; and (**c**) after cooling and constraint removal, and (**d**) after heating for shape recovery. Reprinted from [67].

SMPs are classified into two main types based on their response mechanisms. When it comes to the one-way SME, the change is irreversible; the polymer retains its original shape after the initial programming and requires reprogramming to activate a new shape change. In contrast, the two-way SME is reversible, enabling the material to switch back and forth between two distinct shapes without additional reprogramming steps. The ability of SMPs to recall multiple temporary shapes categorizes them as dual or multiple, underscoring their versatility and potential for various technological applications, as shown in Figure 3 [67]. Thus, SMPs are particularly promising for applications in soft actuators, sensors, artificial muscles, and soft robotics, while SMGs also have several important applications, which are discussed in the next section.

### 2.4. Shape Memory Hydrogels

**Figure 4:**
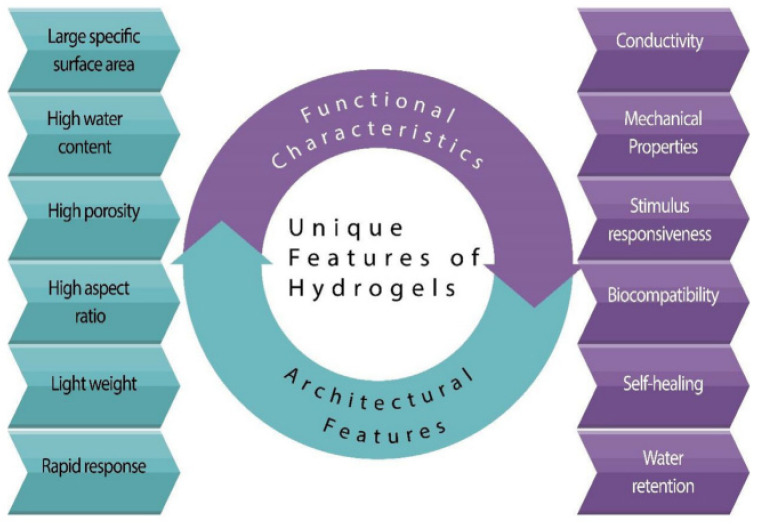
Distinct features of hydrogels. Adapted with permission. Adapted with permission from [50].

A hydrogel is a three-dimensional network of polymers capable of absorbing and retaining significant amounts of fluid, a phenomenon known as swelling. This swelling behavior is induced by physical or chemical crosslinking of the polymer chains that form the network structure. Natural hydrogels are derived from polymers from natural sources such as plants and animals, including cellulose, alginate, chitosan, and gelatin, which maintain the integrity of the hydrogels [68]. Hydrogels can also be synthesized using synthetic polymers such as polyethylene glycol (PEG), polylactic acids, polycaprolactone (PCL), and polyvinyl alcohol through chemical polymerization [69,70,71,72,73]. Shape memory hydrogels (SMHs) have garnered attention because of their inherent biocompatibility, ability to change shape programmatically, customizable physicochemical properties, and controllable biodegradability, as shown in Figure 4 [50]. SMHs are also incorporated with different nanomaterials (fullerenes, nano-onions, nanodots, nanodiamonds, nanohorns, nanotubes, and graphene) to modify structures and morphologies for biomedical applications [74].

These characteristics make SMHs particularly suitable for biomedical applications, where properties such as high stretchability, transparency, ionic conductivity, wettability, and biocompatibility are crucial [75,76,77,78]. SMHs offer innovative solutions in the biomedical field by responding to various biocompatible stimuli, such as thermal, chemical, electrical, and light stimuli, under physiological conditions. Applications include controlled drug delivery through adjustment of the polymer composition, surgical tools such as stents and catheters in which the polymeric network is reshaped, and implantable scaffolds with programmed dimensions for regenerative therapies [79]. Smart hydrogels have gained significant attention for their potential use in drug delivery systems due to their excellent stability, favorable physicochemical properties, and biocompatibility. These qualities make them ideal candidates for enhancing the effectiveness and precision of drug delivery [80]. Different types of SMHs are categorized based on the external stimuli that trigger their actuation mechanisms, including heat, light, moisture, pH, mechanical forces, and electrical and magnetic fields [81,82,83,84,85,86,87]. This diversity underscores the versatility of SMHs in responding to specific environmental cues for targeted biomedical engineering [88]. As mentioned, SMs (alloys, polymers, and gels) are a class of materials that can return to their original shape after memorization through two transformation stages. Considering the primary attributes of SMPs, SMAs, and SMHs, it is clear that SMHs offer an excellent combination of several features including biodegradability, self-healing, and biocompatibility, compared to the congeners (SMPs and SMAs). SMs’ properties are also dependent on their structure and the printing techniques used.

## 3. Printing Techniques for Fabricating SMs

This section reviews the main processes for producing smart materials and the underlying operating principles, advantages, and disadvantages. Smart materials are particularly important in the field of 4D printing because they enable the creation of 3D-printed objects that can change their shape or function over time, which will be discussed in details in next section [89].

### 3.1. Three-Dimensional and 4D Printings

**Figure 5:**
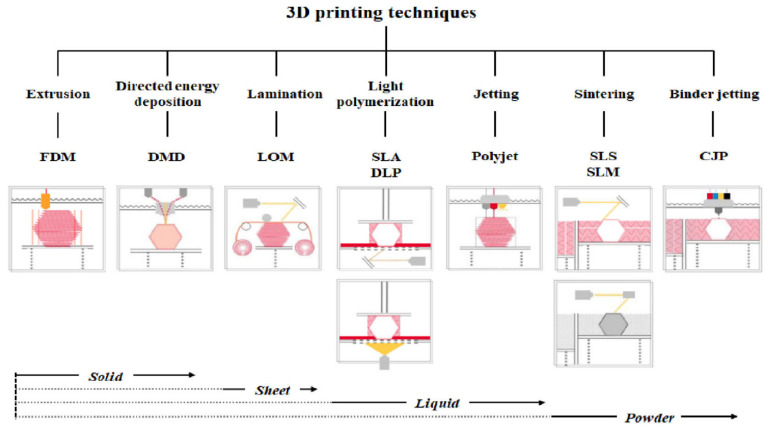
Three-dimensional printers are classified according to type and material. Reprinted from [92].

In 3D printing, materials are deposited and patterned in a drop-on-demand manner, which is often regarded as a bottom–up manufacturing strategy [90]. This enables the quick design and production of numerous smart actuator-based products [91]. The two types of 3D printing procedures are contact-based and contactless. Three-dimensional printing techniques can be classified according to the printing process and material types (Figure 5) [92]. Powder bed fusion (PBF), direct energy deposition, and photopolymerization are typical contactless 3D printing technologies, whereas fused deposition modeling (FDM), material jetting (MJ), and direct ink writing (DIW) are contact-based approaches [93,94,95,96]. Stereolithography (SLA) and FDM are the most commonly used techniques. High-temperature nozzles are used in FDM to feed the filament and subsequently deposit sheets of molten material at high fabrication speeds in a layer-by-layer manner. The significant benefits of FDM include its affordability and adaptability to all sizes of construction, as well as its lower cost compared with other 3D printing methods. In the case of DIW, a wide range of DIW inks can be applied to any substrate of any shape, even those that are complicated or random. Therefore, some researchers consider DIW a potent method for creating precise and complex electronic devices. The significant disadvantages of FDM include the potential for needle clogging at moderate speeds and under strong shear forces [97]. Fused filament fabrication (FFF) is regarded as the most straightforward and popular 3D printing method for a wide range of thermoplastic materials. FFF can be performed at a reasonable price and enables multimaterial 3D printing for diverse applications. SLA is another widely used 3D printing technique. By using a laser to scan liquid UV-curable matter, SLA can be customized and used to print complicated geometries via the photopolymerization step method [98]. This allows for exceptional speed and high print resolution, possibly faster than that of FDM. Additionally, SLA is a perfect fit for the manufacturing of customized soft robotics for wearable applications. The focus of researchers has shifted to micro- and nano-printing methods, such as two-photon polymerization (2PP), also known as direct laser writing (DLW) [99], because of growing miniaturization and the greater need for microfabrication. This method affords outstanding spatial resolution in the 100 nm range upon the exposure of photoreactive resin to high-energy femtosecond laser beams [100]. In 2013, researchers made significant advancements in 3D printing technology by incorporating shape-morphing features into 3D-printed products, which they referred to as 4D printing [101,102]. Therefore, as the 3D printing process advances, new opportunities for 4D printing have emerged. Commercial 3D printers, smart materials, and environmental stimuli such as light, temperature, pH, humidity, magnetic fields, and electric fields have all contributed to the rapid growth of 4D printing [103]. Higher freedom and flexibility in terms of printable geometries can be realized by 4D printing [104]. Additionally, the product’s blueprint is integrated into a flexible, intelligent material through 4D printing. The term “4D” describes dynamic structures derived from conventional 3D-printed structures, denoting the ability of the printed structure to alter at least one of its fundamental characteristics (design, color, property, or functionality) over time via polymerization [105]. This introduces a new frontier for various applications, spanning soft robotics [106], shape memory systems [107], advanced actuators [108], tissue engineering [109], targeted drug delivery [110], cell-loaded constructs [111], and self-deploying aerospace structures [112]. Four-dimensional printing offers a broad scope of multifaceted capabilities, including SMEs, rapid and intricate deformations, adaptable structures, actuation, and sensing in response to the environmental stimuli of SMs.

### 3.2. Three-Dimensional and Four-Dimensional Printing of SMs

**Figure 6:**
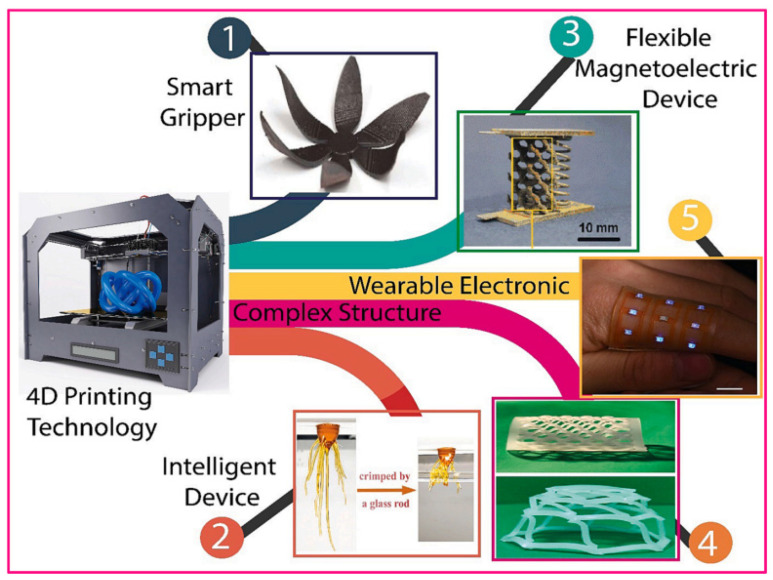
Recently, 4D printing technology was used for various advanced sensors and robotics applications. (1) Smart grippers; (2) intelligent devices; (3) flexible magnetoelectric devices; (4) complex Kirigami-inspired structures; and (5) wearable electronics. Reprinted from [126].

Combined with current 3D printing methods, smart or stimuli-responsive materials, such as thermosets, thermoplastic polymers, and other biomaterials, are just a few of the subtypes of smart materials that can be created using advanced 4D printing. Polylactic acid (PLA) [113], polyvinyl alcohol (PVA) [114], polycaprolactone (PCL) [115], polyurethane (PU) [116], and hydrogels [117] are among the primary smart materials used for creating highly responsive soft devices at both the macro and micro levels [118,119]. Innovative features such as self-adaptation to the environment, self-sensing, and self-healing have been introduced in smart devices via 4D printing. Ren et al. [120] created a highly adaptable smart tactile sensor using 4D printing with shape memory PU and nanocarbon black/PLA composites. The sensors exhibited a distinct change in the sensitivity and measurement range through manipulation of the electrode height and spacing, resulting from the deformation of a SMP during heat treatment. Shape-changing tactile sensors can enable self-adjustment and self-adaptation to achieve human–robot cooperation in sensing. Four-dimensional printing currently uses a variety of novel materials, including liquid crystal elastomers (LCEs) and other hydrogels [121,122,123,124,125]. Four-dimensional printing technology can fabricate a smart sensor with an adjustable measuring range, sensitivity, and shape adaptation, enabling it to sense touch on unstructured objects. For actuator applications, dynamic Diels–Alder (DA) crosslinks, azobenzene, and supramolecular interactions are combined to make 4D-printable liquid crystal elastomers. The development of 4D printing for smart-device applications is illustrated in Figure 6 [126].

### 3.3. Three-Dimensional Printing of Smart Hydrogels

Because of the developing applications in a variety of industries, such as manufacturing, robotics, aerospace, and biomedicine, the 3D and 4D printing market is expanding quickly. It is anticipated that the value of the 4D printing market will reach approximately USD 2.7 billion by 2025, expanding at a compound annual growth rate of more than 20% starting in 2020. A significant driver of 4D printing is the biomedical industry, especially for uses like implants, prosthetics, and medical equipment that need to be self-healing or adaptive. By 2025, the worldwide tissue engineering industry alone is expected to grow to a value of USD 70 billion, with a strong interest in employing 3D and 4D printing technologies to produce bioactive scaffolds that can adapt to changing environmental conditions. With an emphasis on biomedical applications, a number of research industries and institutes are making significant investments in 3D and 4D printing technologies; for example, the MIT Media Lab has been creating 4D-printed devices with responsive materials like SMGs for potential applications in bioelectronics and tissue engineering. The growing need for flexible, self-healing, and responsive materials in tissue engineering, drug delivery systems, and medical devices is driving the integration of SMGs with 4D printing, which has the potential to revolutionize biomedical sectors. With the 4D printing market growing rapidly and biomedical applications continuing to expand, SMGs are becoming a promising material for next-generation technologies in healthcare.

Various polysaccharide-based hydrogels, such as hyaluronic acid, chitosan, and alginate, as well as synthetic hydrogels, such as fibrin, gelatin, and silk fibroin, have been successfully employed in a range of 3D and 4D printing applications [127,128,129]. Stimuli-responsive hydrogels are a family of stimuli-responsive materials used in 4D printing [130,131]. These hydrogels can be utilized to create various structures with remarkable controllability and adaptability because they react to heat, magnetic fields, pH changes, and electric fields [132,133,134]. It might be difficult to quickly prototype hydrogels to create highly tailored and complicated structures using conventional fabrication methods such as casting and electrospinning. Innovative AM provides an excellent opportunity for creating 3D permanent shapes, with enormous potential in the development of intelligent structures. By using stimuli-responsive materials, particularly SMHs, 3D printing can be used to create 3D multifunctional items. The applications of SMH-based 3D-printed devices include smart actuation, drug delivery, tissue engineering, and microgripping. However, because hydrogels can only be produced under mild conditions, not all 3D printing techniques can be used to manufacture SMHs. This review clarifies the 3D printing methods that are specifically utilized for hydrogel processing. Table 1 summarizes the various 3D printing methods used to print SMHs, along with their benefits and drawbacks.

**Figure 7:**
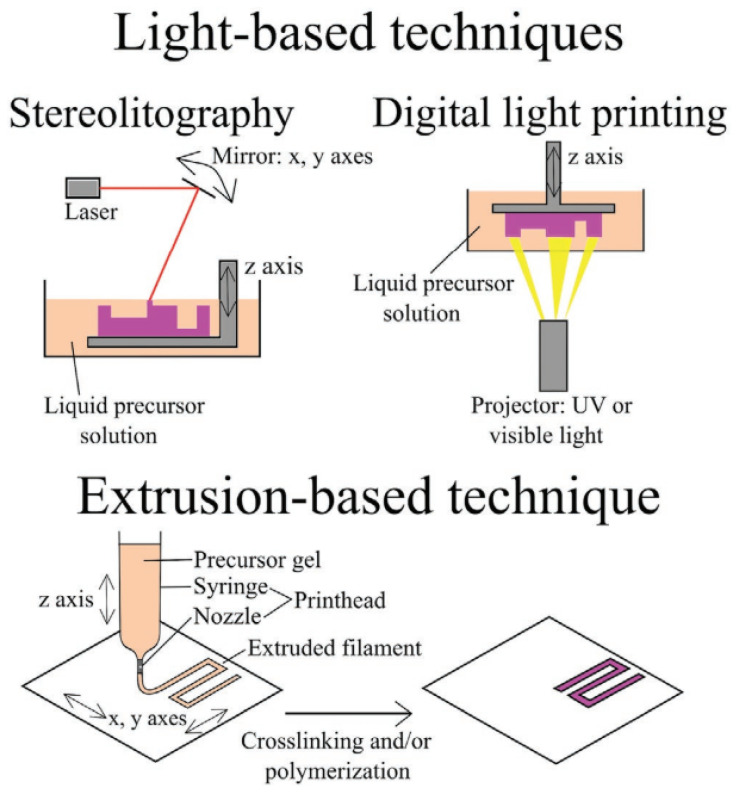
Light-based and extrusion-based printing techniques commonly used in 4D printing of hydrogels. Adapted with permission from [141].

As shown in Figure 7 [141], extrusion-based printing creates 3D structures through the layer-by-layer deposition of molten or semi-molten polymers, dispersions, or pastes on a constructed tray. Extrusion-based printing is further divided into DIW and FDM [142,143,144]. To create 3D objects, FDM melts solid polymers and extrudes them through a nozzle. Despite the various available FDM filaments, their limited functional qualities limit their use in 4D printing. Consequently, the FDM process for 4D printing is extremely difficult because of the lack of intelligent filaments. Ink extrusion and hardening are the processes used in DIW; these are straightforward 3D printing methods. A nozzle can be used to extrude the dispensed fluid ink, which must be hardened immediately after deposition to maintain its structure. Most hydrogel-based structures are created using DIW [145]. The market for plant-based foods is expanding rapidly, spurring the development of stimuli-responsive hydrogels. SLA and TPP exhibit distinct capabilities in terms of resolution, as SLA achieves resolutions between 25 and 100 µm in the x, y, z directions, depending on the optical system and resin properties [146]. This resolution is sufficient for applications like prototyping or functional part production; however, it remains constrained by the single-photon polymerization mechanism, limiting its use in applications for which nanoscale features are required. Compared to SLA, TPP offers a resolution in the sub-micron range, with achievable feature sizes down to 100 nm or smaller [147,148] which is attributed to nonlinear two-photon absorption, confining the polymerization reaction to the focused region of an ultrafast laser beam. SLA is also well known for its scalability, which enables the fabrication of parts exceeding dimensions of one meter, which makes SLA a preferred choice for manufacturing medium-to-large components in the automotive and aerospace industries. TPP faces significant limitations in scalability compared to SLA due to its time-consuming and voxel-by-voxel fabrication process [149]. SLA provides a practical balance between resolution and scalability, making it versatile for a wide range of applications where TPP’s unmatched precision comes at the cost of limited scalability, restricting its adoption to niche applications. The use of SMH materials in 3D printing is another cutting-edge process for creating dynamic structures. Many types of 3D-printed SMHs are indispensable for scaffold formation in tissue repair [150].

#### 3.3.1. Three-Dimensional Printing of Electro and Magneto-Responsive Hydrogels

**Figure 8:**
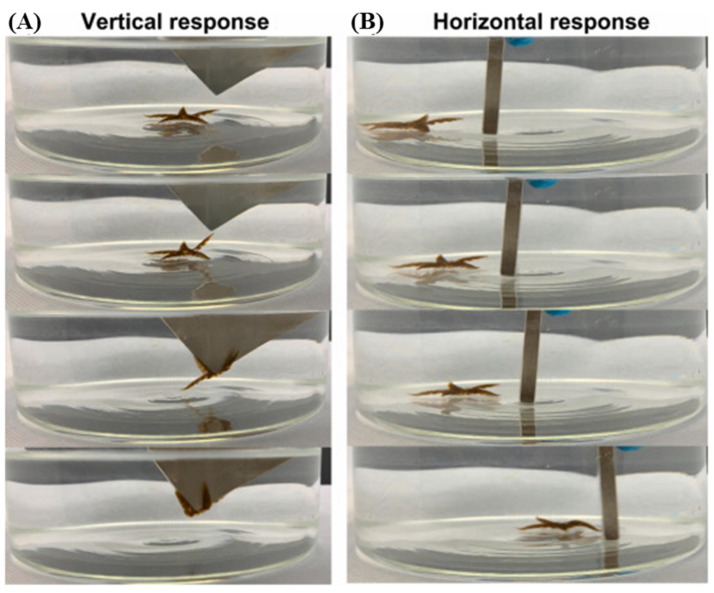
Three-dimensional-printed magnetic starfish hydrogels with 1 wt% SPIONs demonstrating response to a magnet. (**A**) The arms swing towards the magnet and attach to it. (**B**) The hydrogel starfish can follow the movement of the magnet. Reprinted from [149].

**Figure 9:**
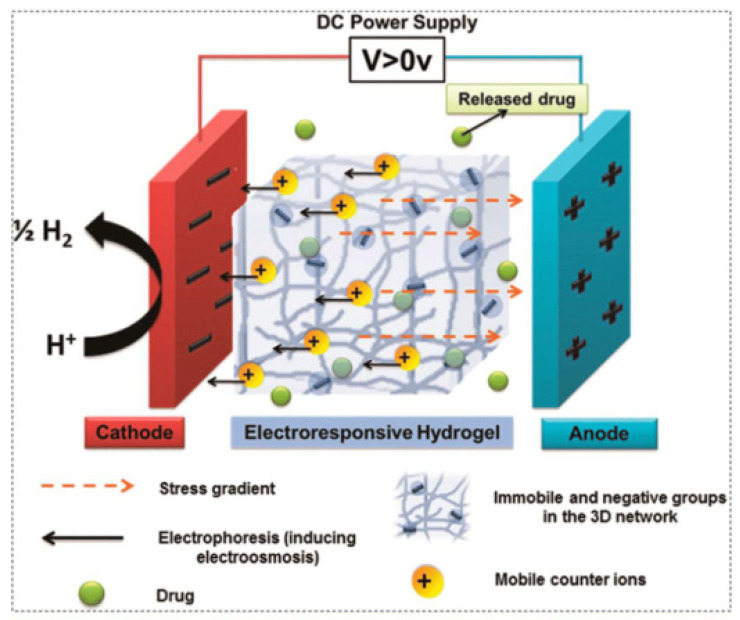
Schematic illustration of the mechanisms for electro-induced hydrogel swelling for drug delivery applications [152]. Reprinted with permission.

Several recent studies have been conducted on 3D-printed magnetically powered actuators with programmable structures and intricate geometries. Specifically, using 3D printing to build magnetic hydrogels opens up possibilities for hydrogel actuators that can be controlled remotely and can be used for navigation [151]. Mohammad et al. [149] used 2 wt% of superparamagnetic iron oxide nanoparticles (SPIONs), along with acrylamide and PEGDA, to 3D print starfish hydrogels that exhibited 10% shape deformation upon swelling. The starfish, as seen in Figure 8 [149], clung to a magnet with all of its arms when exposed to a magnetic field stimulus. Upon removal of the magnetic stimulus, the printed hydrogels resumed their original formation and maintained their remarkable forms. These hydrogels have potential use in soft robotics and magnetically activated actuators. Using MWCNT/polydimethylsiloxane (PDMS) composites, Wang et al. [152] printed a millimeter-scale magnetic soft robot. Moreover, rGO/PDMS composites printed directly onto a neodymium–iron–boron (NdFeB)/PDMS composite substrate were used to construct a dual-sensor configuration-based magnetic soft robot. Electro-responsive SMGs are the class of smart materials which undergo a physical change in form of shrinkage, swelling or shape changes by an applied external field [153,154,155]. Electro-responsive SMGs includes synthetic (e.g., Polyaniline, polypyrrole, sulfonated styrene, polythiophene, and polyvinyl alcohol) and natural materials (e.g., chitosan, alginate, and hyaluronic acid [155]. The actuation behavior of electro-responsive SMGs under electrical stimuli can be governed by force mosmotic pressures that are influenced by forces such as polymer–polymer affinity, ionic pressure, and rubber elasticity. Disruption of the balance between these forces leads to swelling and deswelling in electro-responsive SMGs when an electrical field is applied. This occurs in an aqueous medium of H+ and OH− ions along the polymer chains Figure 9 [152]. The volume transition in electro-responsive SMGs is induced by the osmotic pressure difference between the SMGs and the surrounding aqueous solution. This osmotic pressure difference serves as the driving force for controlled drug release from the electro-responsive SMGs. These achievements demonstrate the potential of magnetic shape memory materials for biomedical and microfluidic engineering applications.

#### 3.3.2. Three-Dimensional Printing of Temperature-Responsive Hydrogels

Over the years, temperature sensors have attracted considerable attention because of their quick and highly regulated reactions [156], which result in lower chemical residues and increased biosafety [157]. For example, *N*-isopropyl acrylamide hydrogel-based thermosensitive valves are highly effective in regulating fluid flow in micropumps [158]. The adaptability and high performance of hydrogels derived from their multifunctionality and self-healing properties are now widely recognized owing to research on various biomaterials and innovative nanomaterials. For instance, Wu et al. [159] created a self-healing hydrogel based on PU and gelatin, which could be 3D-printed, exhibiting remarkable photo- and thermo-responsive activity for a week without freezing. Temperature-responsive graphene oxide (GO)-enhanced poly (*N*-isopropyl acrylamide) (PNIPAM) hydrogels are promising materials for wound dressing applications [160]. As demonstrated by Gao et al., the compressive strength of the hybrid hydrogels is 49 times greater than that of PNIPAM, and the hybrid hydrogels are highly adjustable, with transition temperatures between 32.7 and 34.8 °C.

#### 3.3.3. Three-Dimensional Printing of pH-Responsive Hydrogels

pH-responsive 3D-printed hydrogels can be used to create live, dynamic structures [161]. For example, Zhang et al. [162] prepared a predesigned four-armed shape that was strongly attached to an Ecoflex mold to study SMHs for small-scale soft grippers. Zhang et al. showed that under different stimuli, the SMHs grippers autonomously recorded logical output information equivalent to logic gates (AND or OR gates) triggered by expansion or contraction. The proposed method shows great potential for regulating the movements of small soft robots and other machinery under the effect of stimuli. Digumarti et al. [163] examined 3D-printed konjac glucomannan (KGM)–borax-based hydrogels that exhibit self-healing and responsive features for soft robotic applications. KGM–borax demonstrated 98% self-healing effectiveness in underwater environments. Therefore, 3D-printed KGM–borax-based hydrogels are ideal for next-generation soft robots that can respond to different environmental stimuli and are sufficiently durable to withstand adverse conditions. By using SLA-based 3D printing, Odent et al. [164] created anisotropy-encoded poly(2-carboxyethyl) acrylate (PCEA), PNIPAM, and poly(2-carboxyethylacrylate) hydrogel actuators. The multi-responsive hydrogel-based actuators underwent rapid and repeatable shape changes. Temperature- and pH-based stimuli also induced continuous bidirectional bending of the soft actuators, making them suitable platforms for gripping and releasing various items.

#### 3.3.4. Three-Dimensional Printing of Humidity-Responsive Hydrogels

Water- and humidity-responsive hydrogels undergo basic shape-morphing behaviors such as swelling and deswelling through the absorption and evaporation of water vapor, respectively [165]. This complex behavior is linked to the crosslinking density of the hydrogel, where the volumetric expansion is reduced at high crosslinking densities [166]. Water-responsive hydrogels can be combined with cutting-edge additive printing techniques to create intricate, programmable, and complex shape-controlled objects. Therefore, 3D printing using PNIPAM, PEGDA, poly(acrylamide) (PAAm), poly (butyl methacrylate), and PAA has been extensively studied and documented with regard to its use for producing unique forms, which are currently in high demand [167]. For example, Yang et al. [168] presented a regulated approach using a single-material printing technique to achieve humidity-responsive programmable deformations of PEGDA hydrogel architectures. Variations in the water absorption and swelling properties of the PEGDA hydrogels and controlled bending deformation of the structures were achieved. Additionally, the micromanipulator could correctly grasp and release minuscule items measuring centimeters or even millimeters. The proposed design presents a novel concept for efficient utilization of microrobots in tissue engineering and medication delivery. These intriguing findings may help maximize the swelling response of water-responsive hydrogels to several stimuli such as pH and temperature.

#### 3.3.5. Three-Dimensional Printing of Solvent-Responsive Hydrogels

Under the influence of organic or inorganic solvents, the structure of solvent-responsive hydrogels can be transformed from a fixed to a transient shape. Ethanol, citric acid, and acetone have been extensively studied as potential solvent stimulants. Three-dimensional printing techniques can improve the complex shape-morphing behavior of structures with sophisticated designs constructed using hydrogel systems [169]. Solvent-responsive hydrogels, as opposed to those responsive to conventional stimuli, offer special advantages for a wide range of functionalities when printed hydrogel architectures are constructed [170]. Consequently, solvent-responsive hydrogels can be applied in numerous fields such as tissue engineering and soft robotics. To create self-rolling bilayer films, Cao et al. [171] explored the use of ionic-responsive sodium alginate methacrylate (SAMA) and thermo-responsive polycaprolactone dimethacrylate (PCLDMA). Complex structures were fabricated using DIW-based 3D printing. The printed bilayer displayed controllable self-rolling behavior when immersed in warm water and a Ca^2+^ solution. Furthermore, the application of 4D printing to these biomaterials has great potential for developing vascular stents [171]. The structures exhibit intricate and sophisticated shape-morphing behaviors because of the ability of organic solvents to create a reversible dilation mechanism and constrict the polymer matrix.

## 4. Applications of Printed Smart Materials

### 4.1. Soft Robots

Owing to its unique position in fascinating applications such as sensors, actuators, and tissue engineering, soft robotics has consistently attracted significant attention from academia and industry [172]. Soft robotics is an attractive option for developing highly flexible and adaptive sensors for use in complicated environments, owing to its flexible actuation which is triggered by numerous methods. Soft robotics is meant to mimic the extraordinary shape-morphing behavior of natural creatures and to enable highly elastic, motorless-driven processes, in contrast with those achievable with their rigid counterparts [173]. Three-dimensional-printed soft robots composed of flexible polymeric materials such as hydrogels offer increased versatility through capabilities such as bending, rolling, twisting, and folding. These attributes are particularly advantageous for navigating small spaces and complex environments where direct human interaction is impractical [174]. Moreover, these soft robots can be equipped with advanced tools such as enhanced 3D vision, highly dexterous surgical instruments, and intuitive human–robot interfaces. These innovations can significantly enhance the precision of tool manipulation, facilitating their successful application in minimally invasive surgery, thereby augmenting surgeons’ capabilities. For example, Sun et al. [175] printed thin-walled watertight and pressurized structures to create marine-sourced hydraulic actuators using new calcium alginate hydrogels. The actuators had multiple characteristics, including intricate forms and interior spaces that were safe for marine life, and were edible, biodegradable, and digestible. Finally, the marine robot displayed sophisticated soft-device capabilities, such as the ability to move various objects. Takishima et al. [176] used a 3D-printed hydrogel that resembled a jellyfish to produce a unique soft actuator. The hydrogel actuator parts were extremely elastic, and their normalized contraction ratios were nearly identical to those of moon jellyfish. The 3D-printed actuator is promising for a robot that mimics jellyfish.

### 4.2. Tissue Engineering and Drug Delivery

**Figure 10:**
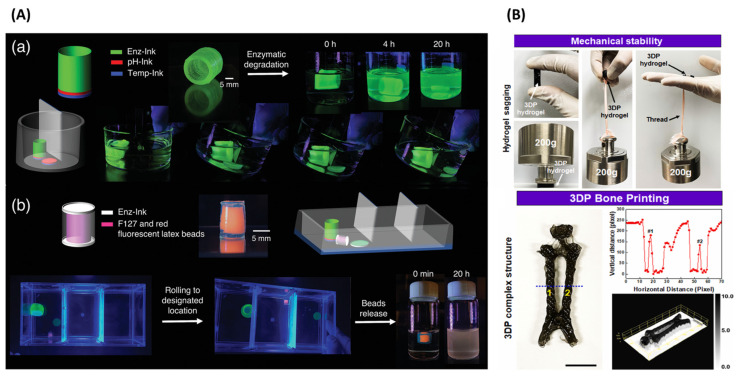
(**A**) Cargo transport and delivery would expand their applications in the broader drug delivery field (**a**) 3D printed cylinder using Temp-Ink, pH-Ink, and Enz-Ink; two cylinders are shown, one before enzymatic degradation with proteinase K and the other after degradation by the enzyme which allowed it to pass through a gap under the barrier, (**b**) An enzymatically degradable cylinder filled with red fluorescent latex beads can roll into a designated location and release red fluorescent latex beads in the presence of proteinase [reprinted with permission from Ref. [176]. (**B**) Weightlifting study to evaluate the mechanical stability of the 3D-printed hydrogel (the 3D printing was performed using 90% infill density to maximize the mechanical performance), and demonstration of a full-length rat bone structure using GelMA-PPy ink. Adapted with permission from [177].

The growth of 3D-printed SMHs is driven, in part, by their utility in drug delivery systems [177]. A 3D-printed protein-based programmable structure composed of hydrogels that can change in response to several stimuli, including pH, temperature, and an enzyme, was studied by Narupai et al. [178]. PNIPAM and poly (dimethylaminoethyl methacrylate) hydrogels were used in DIW-based 3D printing. As shown in Figure 10A, the protein-based hydrogels exhibited remarkable shape changes in response to multiple stimuli and were irreversibly changed by enzymatic breakdown. The ability of hydrogels to autonomously change their shape and size offers significant advantages for cargo delivery and release. By combining different inks, researchers fabricated a cylindrical structure with bilayer disk made of Temp-Ink and pH-Ink for the bilayer disk base, and Enz-Ink for the body of the cylinder (Figure 10A). This cylindrical hydrogel was placed into a container with a narrow channel in the center. Initially, the cylinder was physically trapped on the left side of the container because it was too large to pass under the barrier. To demonstrate the release of cargo, researchers used Enz-Ink to fabricate an enclosed cylinder filled with a solution of red fluorescent latex beads (Figure 10A). Protein-based hydrogels are promising for application in controlled drug delivery systems.

Considerable advancements have been achieved in the 3D bioprinting of conductive hydrogels, enabling the generation of biomimetic structures with progressive complexity and high resolution. Customizable physicochemical properties, sensitivity to various stimuli, biodegradability, and remarkable biocompatibility for particular medical treatment situations are only a few of the distinctive features of 3D-printed hydrogels. DIW-based, extremely stable, and conductive polypyrrole (PPy)-grafted GelMA structures were proposed by Dutta et al. [179]. The hydrogel with triple-crosslinking exhibited excellent shear-thinning characteristics. The high-resolution biological architecture exhibited various characteristics, including minimal disruption of the “plug-like non-Newtonian” flow behavior, high structural stability, and self-support, as shown in Figure 10B. Before demonstrating the fabrication of complex biological structures, the mechanical stability of the 3D-printed constructs was tested by using a weightlifting method, as shown in Figure 10B. The 3D-printed and crosslinked hydrogel was able to withstand a load of 200 g, demonstrating the durability of the fabricated sample. Figure 10B further illustrates that the hydrogel ink successfully printed the bone construct according to the designed structure.

### 4.3. Sensors

Ideal sensing materials should integrate numerous actuation and sensing functions. SMHs are useful in various soft devices, including flexible electronics, when printed in three dimensions. Wu et al. [180] prepared a printing ink using a hydrogel based on poly (acrylic acid (AA)-N-vinyl-2-pyrrolidone (NVP)) and CMC. The printed hydrogel exhibited exceptional mechanical qualities and self-healing traits, such as a healing strain of 91% and healing stress of 81%. Using photocurable 3D printing, these innovative hydrogels were successfully applied in various customized products (such as manipulators) with complicated structures, high durability, and multifunctionality. Therefore, high-performance hydrogels manufactured in 3D form can potentially be applied in flexible wearable sensors. Using polydimethylsiloxane (PDMS) and silver nanoparticles (NPs), Khoshnoo et al. created a unique 3D-printed sensor device for real-time pH monitoring. Multilayer printing of sensors and reusable electronic/communication circuitry was made possible by 3D-printed nanomaterials on skin-like flexible substrates. These sensor systems exhibited outstanding biocompatibility, high sensitivity, and mechanical flexibility across several pH ranges. The printed sensors enabled real-time pH monitoring in an ex situ hydrogel-based wound model [181]. Recently, ionotropic hydrogels have attracted considerable attention for the fabrication of flexible electronics, wearable technology, and energy devices. Consequently, a range of wearable healthcare, biological, and food technology applications is now possible.

### 4.4. Food Industry

The market for plant-based foods is expanding rapidly, spurring the development of stimuli-responsive hydrogels [182,183,184]. Hydrogels provide several benefits, including the ability to achieve tailored nutrition, food that meets consumer preferences and flavor requirements, and improved functionality and texture of vital plant-based components. Moreover, hydrogels are cost-effective and facilitate the efficient use of various food ingredients. Therefore, a wide range of natural polysaccharides and 3D-printed hydrogels has been investigated [126]. Moreover, the food industry is currently expanding its boundaries through 3D printing technology using naturally occurring hydrogels. Currently, 3D printing is used to create intricately designed meals targeted mainly at the young and elderly [185,186,187,188]. Foods produced using 3D technology can maintain essential nutrients for consumption, enabling facile ingestion by patients and the elderly population.

### 4.5. Electromagnetic Interference (EMI)

In addition to food technology, 3D printing has been used for electromagnetic interference applications. EMI is employed to prevent interference during the downsizing of electronic and telecommunications devices [189]. These types of equipment for the aircraft and defense sector can be produced with great success using 3D-printed “intelligent” materials and “smart” structures [190]. Several researchers have attempted to create EMI materials by combining polymers and hybrid hydrogels in smart architectures. For EMI shielding, Menon et al. [191] created 3D-printed polypropylene (PU) structures with silver coverings for memory applications. The remarkable shape memory capabilities of PU were further enhanced by electroless deposition and polydopamine (PDA) coating. Thus, the materials can potentially be used in EMI applications for shape memory-triggered actuators. Liu et al. [192] created unique Ti3C2-MXene-functionalized conductive hydrogel inks for 3D printing in a notable breakthrough. The proposed MXene/poly(3,4-ethylenedioxythiophene) and polystyrene sulfonate (PEDOT: PSS) hydrogels exhibited remarkable mechanical properties, including fatigue resistance, outstanding stretchability, and flexibility. This innovation opens opportunities for creating printed hydrogels for individualized EMI shielding applications. Security printing, intelligent food packaging, and printing applications are promising areas for this endeavor.

## 5. Summary, Challenges and Future Work

This review provides a comprehensive understanding of different SMs, fabrication techniques to print SMs, various mechanisms of action exhibited by SMs, their applications in various fields, and potential future directions. In this review, we further explained and focused on SMGs, fabrication techniques to print SMGs for different applications. SMGs were also summarized with respect to their external stimuli along with their applications. This review also outlines some of the major production bottlenecks in printing devices with certain curvatures and hanging structures, mainly using DIW, where structural collapse due to gravity is a limitation. A lot of work has gone into creating innovative composite frame–membrane constructions that exhibit shape-morphing behavior in response to stimuli for the creation of untethered soft actuators that can detect motion. Three-dimensional- and four-dimensional-printed SMs applications are still in their infancy and are being widely used to create food, actuators, sensors, soft robotics, biomedical, and EMI shielding.

There are several challenges associated with printing SMs, specifically when combining SMGs and 4D printing technologies such as hydration sensitivity, complex actuation, resolution, layer bonding, degradation and large-scale 4D printing of SMGs. The hydration sensitivity and complex actuation of SMGs are significant hurdles, as SMGs often respond unpredictably to changes in moisture and humidity during the 4D printing process. In addition, achieving rapid and predictable actuation of materials with 4D printing is difficult due to the complex interactions of printed shape memory gel structures. Issues associated with the limited resolution and quality of 4D printing materials often arise, along with poor adhesion and compatibility between printed SMGs and other materials, without compromising the responsive characteristics of the printed SMGs. SMGs may degrade or lose their shape memory properties over time, which can result in performance degradation in 4D-printed systems, making printed SMGs less reliable for long-term applications. Moreover, 4D printing technologies capable of processing SMGs are not as widespread or as affordable as traditional 3D printing methods. After 4D printing of SMGs, the initial activation phase is challenging and may require specific curing, drying, or stabilization steps for the post-processing phase of 4D printing. When it comes to large-scale or industrial 4D printing, several limitations exist that can impact both the performance of SMGs and the scalability of the technology. SMGs can be challenging to process using traditional 4D or 3D printing procedures due to their softness, the specific moisture level they require, and the difficulty in reliably controlling the gel’s viscosity, stability, and handling during large-scale printing. The speed and precision of 4D printing with SMGs may not yet be sufficient for large-scale manufacturing, as it is challenging to regulate how printed SMGs react to stimuli across large structures, resulting in irregular shape changes that reduce efficiency in industrial applications.

The development of printing is hampered by the poor shape-fidelity of printed patterns and poor printability of very soft materials such as SMGs, which makes it difficult to 3D print complicated hydrogel structures. However, these difficulties can be overcome by improving the rheological properties of the hydrogels. Investigations of the rheological properties and biocompatibility of hydrogel materials for 3D printing are necessary to prevent immature responses and uncontrollable triggering caused by unintended stimuli. Computational models will become more capable of optimizing and enhancing the performance of printed materials as printing processes become more intelligent and machine learning-based. Given the considerable scholarly interest in this field, emerging frameworks will eventually be used to design structural components that can function adequately and satisfy the requirements of real-world applications. The incorporation of Artificial Intelligence (AI) into the printing processes is an emerging trend that could improve manufacturing, boost performance, and increase the functionality of SMGs, especially in biomedical applications. AI has the potential to revolutionize SMGs manufacturing by addressing the issues of material shrinkage and warping during 4D printing, accuracy, personalization, and productivity. Technological advancements in intelligent and active structures are expected to significantly influence the design of materials for practical applications in the near future.

## Figures and Tables

**Table 1:** Summary of shape memory materials (SMMs) comparison.

Printing Technique	Material	Properties	Applications	Ref
μSLA	PNIPAM/PEGDA/AA	Bending	Microrobots	[107]
SLA	Chitosan/PEGDMA/CNF	Folding	Tissue scaffolds	[108]
Extrusion	GelMA/rGO	Bending	Bone tissue scaffolds	[135]
DLP	PEGDMA	Folding	Microfluidic devices	[136]
DIW	AA/CNC	Self-healing	Wearable sensors	[137]
FDM	PU/elastomer	Folding and twisting	Origami structures	[138]
Vat photo- polymerization	PNIPAM/laponite nanoclay/NdFeB	Folding and bending	Soft millirobots	[139]
TPP	PEGDA	Swelling	Biosensors	[140]
